# Modeling the impacts of influenza antiviral prophylaxis strategies in nursing homes

**DOI:** 10.1017/ash.2023.234

**Published:** 2023-09-29

**Authors:** Casey Zipfel, Sinead Morris, Prabasaj Paul, Matthew Biggerstaff, Rachel Slayton

## Abstract

**Background:** Antiviral chemoprophylaxis for influenza is recommended in nursing homes to prevent transmission and severe disease among residents with higher risk of severe influenza complications. Interim CDC guidance recommends that long-term care facilities initiate antiviral chemoprophylaxis with oral oseltamivir for all non-ill residents living in the same unit following the start of an outbreak in a facility (ie, ≥2 patients ill within 72 hours and of whom at least 1 resident has laboratory-confirmed influenza). Prophylaxis continues for a minimum of 2 weeks and for at least 7 days after the last laboratory-confirmed case. However, facilities may not strictly adhere to this guidance, with 1 study showing up to 68% of facilities were nonadherent to national guidance (Silva et al 2020). Here, we model the potential impacts of different antiviral prophylaxis strategies. **Methods:** We developed a susceptible–exposed–asymptomatic–infected–recovered (SEAIR) compartmental model of an average-sized nursing home comprising short-stay residents, long-stay residents, and healthcare personnel (HCP). Persons treated with antiviral chemoprophylaxis were less susceptible to infection, had a lower probability of symptoms if infected, a reduced viral load, and a shortened duration of infectiousness. We included influenza vaccination for residents and HCP through reduced probability of symptomatic infection. Coverage rates were estimated from CDC FluVaxView and CMS COVID-19 nursing home data. As a base case, we modeled a scenario with prophylaxis implemented according to guidance. We varied uptake by residents and HCP (from 10% to 90%), case thresholds for prophylaxis initiation (1–5 cases identified), and timing of prophylaxis cessation: either time dependent (ie, 10–14 days of prophylaxis) or case-dependent (ie, continuing prophylaxis for 1–7 days with no cases). **Results:** In the scenario based on current guidance, prophylaxis reduced resident cases by 16% and resident hospitalizations by 45%, compared to no prophylaxis (Fig. 1A). Scenarios that differed from the guidance altered case burden and timing: Time-dependent prophylaxis cessation increased resident cases and hospitalizations (Fig. 1A). Timing of prophylaxis initiation had slight effects on the timing of the epidemic and minimal effects on resident cases and hospitalizations (Fig. 1B). High resident uptake was important for reducing resident cases and hospitalizations (Fig. 1C), but increasing HCP uptake had minimal effect (Fig. 1D). **Conclusions:** Our findings support the current prophylaxis guidance. Promptly implementing prophylaxis reduces resident cases and hospitalizations. Continuing prophylaxis until cases are no longer identified reduces cases and hospitalizations.

**Figure:**
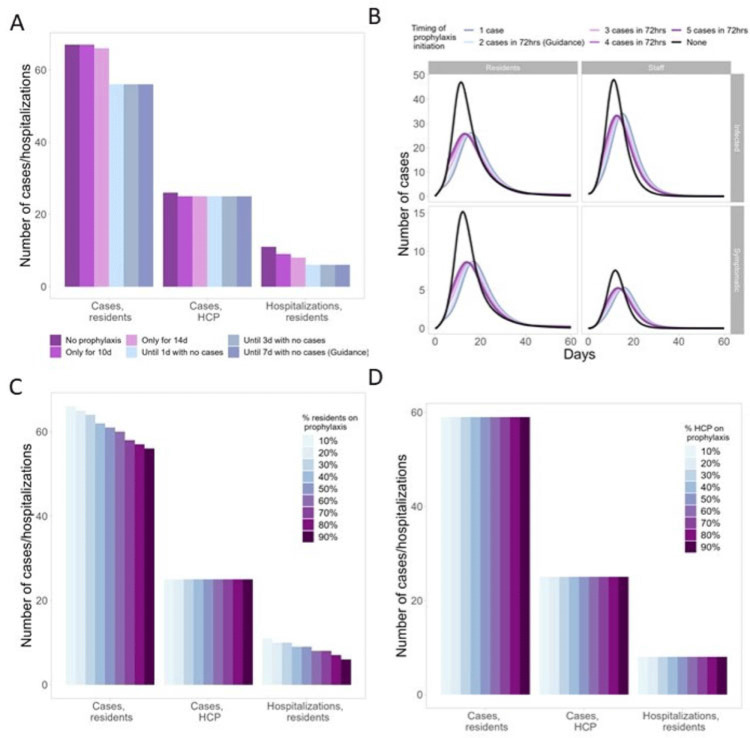

**Disclosure:** None

